# Novel therapeutic strategies for chronic hepatitis B

**DOI:** 10.1080/21505594.2022.2093444

**Published:** 2022-07-06

**Authors:** Sandra Phillips, Ravi Jagatia, Shilpa Chokshi

**Affiliations:** Institute of Hepatology Foundation for Liver Research London UK, School of Immunology and Microbial Sciences King’s College London, London, UK

**Keywords:** Chronic hepatitis B, therapeutic targets, direct-acting antivirals, host-targeted therapies, cccDNA

## Abstract

The last few years have seen a resurgence of activity in the hepatitis B drug pipeline, with many compounds in various stages of development. This review aims to provide a comprehensive overview of the latest advances in therapeutics for chronic hepatitis B (CHB). We will discuss the broad spectrum of direct-acting antivirals in clinical development, including capsids inhibitors, siRNA, HBsAg and polymerase inhibitors. In addition, host-targeted therapies (HTT) will be extensively reviewed, focusing on the latest progress in immunotherapeutics such as toll-like receptors and RIG-1 agonists, therapeutic vaccines and immune checkpoints modulators. A growing number of HTT in pre-clinical development directly target the key to HBV persistence, namely the covalently closed circular DNA (cccDNA) and hold great promise for HBV cure. This exciting area of HBV research will be highlighted, and molecules such as cyclophilins inhibitors, APOBEC3 deaminases and epigenetic modifiers will be discussed.

## Introduction

Chronic Hepatitis B virus infection (CHB) is amongst the biggest threats to global public health [1]. Despite the availability of a preventative vaccine, one person dies from Hepatitis B virus (HBV)-related disease every 30 seconds. The World Health Organisation estimates that 257 million people worldwide live with CHB, and due to a lack of robust epidemiological data, this is widely thought to be an underestimation [2] CHB is associated with long-term morbidity, including liver cirrhosis, and is closely linked to the development of hepatocellular carcinoma (HCC), the 2nd most lethal cancer globally, accounting for 600,000 deaths annually [2].

CHB is a very dynamic process, dependent on the interaction between the virus, hepatocytes, and the host immune response. The natural history CHB is broadly segmented into five phases defined by the following clinical parameters: alanine aminotransferase (ALT), serum HBV DNA, Hepatitis e surface antigen (HBeAg) and Hepatitis surface antigen (HBsAg) (see Table 1). Whilst most patients transition from one phase to the next, some do not go through each phase and may revert to an earlier phase. Importantly, patient management and decisions to treat are based on these phases.

**Table 1.** Natural history of HBV infection divided into 5 phases. +++++: almost always detected; ++++: frequently detected; +++: often detected; ++: occasionally detected; +: rarely detected.

	HBeAg positive		HBeAg negative		HBsAg negative
	Chronic infection	Chronic Hepatitis	Chronic infection	Chronic Hepatitis	
Serum HBV DNA	>10^7^ IU/mL	10^4^-10^7^ IU/mL	<2000 IU/mL	>2000 IU/mL	Undetectable
ALT	Normal	Elevated	Normal	Elevated	Normal
HBeAg	Positive	Positive	Negative	Negative	Negative
HBsAg	High	High/Intermediate	Low	Intermediate	Negative
cccDNA	+++++	+++++	++	+++	+
Integrated HBV DNA	+++	++++	+++	++++	+
Liver disease	Minimal	Moderate/Severe	None	Moderate/Severe	None
Old terminology	Immunotolerant	Immune active	Inactive carrier	Reactivation	

The primary goal of current therapies for CHB is to suppress ongoing viral replication and reduce liver injury to improve patient survival but fail at eradicating HBV infection [3]. However, recent advances in HBV research and the success of curative antiviral treatments for chronic hepatitis C have boosted the development of new therapeutic strategies for CHB, with many promising compounds currently undergoing clinical evaluation. This chapter aims to provide a comprehensive overview of the latest treatment approaches for CHB.

## Goals of therapy for CHB

Experts in the field have recently reached a consensus on defining a therapeutically-induced cure for CHB. Three main definitions have been adopted (also see Table 2) [4]:

1. ***Partial cure*** is defined as the induction of a persistently undetectable viral load and normalisation of ALT with detectable HBsAg levels after finite treatment. This landscape with improvements in virological and biochemical responses reduces the progression of cirrhosis and greatly improve patients’ quality of life and survival. However, despite these clear clinical benefits, the risk of HCC remains.
2. ***Functional cure*** is characterised by a sustained undetectable viraemia with a durable loss of HBsAg with or without seroconversion to HBsAg antibody. This state includes the persistence of covalently closed circular DNA (cccDNA), the template for viral transcription. A functional cure is the clinical end-point for safe nucleos(t)ide analogues (NAs) treatment cessation and is the goal for multiple drugs currently in clinical development. This type of cure is associated with a resolution of ongoing liver injury and a further decrease in the risk of HCC to a level equivalent to individuals experiencing spontaneous viral clearance.
3. ***Complete cure*** is associated with complete loss of circulating and intrahepatic HBV DNA, loss of HBsAg with production of HBsAg antibodies, and eradication of cccDNA. However, the current consensus is that this curative state will be challenging to achieve due to the persistence of cccDNA.
4. ***Sterilisingcure*** is defined by a complete cure together with the removal of integrated HBV DNA fragments from the host chromosomes. This definition of a cure which equates to a state resembling vaccinated individuals who have never been exposed to HBV is highly improbable.

**Table 2.** Definition of HBV cure. ± Anti-HBsAg may be detected.

	Partial cure	Functional cure	Complete cure	Sterilising cure
Serum HBV DNA	Undetected	Undetected	Undetected	Undetected
HBsAg	Detected	Undetected	Undetected	Undetected
Anti-HBsAg	Undetected	±	Detected	Detected
cccDNA	Detected	Detected	Undetected	Undetected
Integrated HBV DNA	Present	Present	Present	Absent

## Standard of care

Since the discovery of HBV in 1965, major advances in understanding HBV biology and its pathogenesis have led to the approval of two classes of drugs to treat CHB: antivirals and immunomodulators such as NAs and Peg-Interferon-α (PEG-IFN-α) respectively [5]. The latest approved NAs, Entecavir (ETV), Tenofovir Disoproxil Fumarate (TDF), and Tenofovir Alafenamide (TFA), are highly potent antivirals with high genetic barriers to resistance and excellent safety profiles [5]. NAs target the reverse transcription activity of the HBV polymerase and, as such prevent the biosynthesis of HBV DNA, which suppresses viraemia to undetectable levels and normalises ALT [6]. Consequently, NAs halt HBV transmission, reduce liver injury and fibrogenesis, lower the risk of decompensation and HBV-related tumorigenesis, thereby limiting the need for liver transplantation [5,7–9]. Unfortunately, the numerous benefits of NAs do not include HBsAg loss – which is rarely achieved in NAs treated CHB patients or, importantly, the elimination of cccDNA. NAs do not target cccDNA, allowing its persistence in the infected hepatocytes (see Table 3). Additionally, the impact of NAs on HBV replication is not absolute. The presence of residual viraemia promotes *de-novo* infection and the replenishment and maintenance of the cccDNA pool. As a result, virological and clinical relapses occur in the majority of patients when NAs treatment is discontinued. Thus, these events define NAs as a life-long therapy which is associated with increasing costs, poor treatment compliance and potential toxicity [5]. Finally, HCC incidence, although significantly reduced on NAs is not eliminated, a result of cccDNA persistence and the integrations of HBV DNA fragments in the host genome.

**Table 3.** Advantages and disadvantages of NAs and PEG-IFN-α in the treatment of CHB.

	Nucleos(t)ides analogues	PEG-IFN-α
Advantages	Oral Good tolerability No Contraindications	Finite duration Higher rate of HBeAg seroconversions (30%) Higher rate of HBsAg loss (5–8%) Loss of cccDNA may occur
Disadvantages	Indefinite duration HBsAg loss <2% No effect on cccDNA	Low tolerability (Significant side effects) Subcutaneous injections

A wide range of studies has established that resolution of HBV infection is effectively achieved through immunological control [10–15]. Long-term NA treatment has been shown to modulate the immune response to HBV in a proportion of patients. Partial recovery of ineffective HBV-specific CD4 and CD8 T cell responses in CHB can occur as a consequence of NAs-induced suppression of viral replication, but this is not complete and often reverts when NA treatment is discontinued [12,13,16–19]. Interestingly, NAs do not restore the antiviral functions of Natural Killer (NK) cells and this compartment may be important to target for the development of strong and durable immunity to CHB.

PEG-IFN-α based therapies are an alternative strategy for CHB (see Table 3). Notably, a finite course of treatment with PEG-IFN-α can result in the loss of HBeAg, a surrogate marker of active replication and can lead to the production of antibodies to HBeAg (HBeAg seroconversion). HBeAg seroconversion in patients who are in the high replicative phase of the disease (HBeAg positive (HBeAg+) CHB) is associated with a low viral replication rate and improvement of clinical outcome. In addition, PEG-IFN-α treatment can also result in HBsAg loss and reduction in cccDNA, leading to a functional cure.

IFN-α is known to elicit the activation of NK cells, which participates in PEG-IFN-α induced control and clearance of HBV infection observed in some patients. It has been reported that PEG-IFN-α therapy could promote the activation and proliferation of cytokine-producing-CD56^bright^ NK cells that produce potent antiviral cytokines [20,21]. However, the frequency of IFN-γ-producing HBV-specific T cells and their proliferative capacity does not improve during the first 6 months of therapy with PEG-IFN-α [22]. Indeed, IFN-α induces a dramatic reduction in the absolute number of HBV-specific CD8 T cells and cytomegalovirus-specific CD8 T cells [21]. This lack of impact on HBV-specific T cells may explain the limited efficacy of IFN-α treatment in CHB. However, the recovery of HBV-specific CD8 T cell responses has been documented in patients who clear infection post-IFN-α treatment, highlighting the importance of an activated adaptive immunity [16,23]. The immunological mechanisms underlying the response to IFN-α are still not well understood and deserve further clarification for treatment optimisation.

PEG-IFN-α is also known for its directly antiviral activities, including suppression of viral replication via the activation of IFN-stimulated genes (ISGs), which block capsid assembly, inhibit HBV RNA synthesis and impair viral transcripts production [24–31]. IFN-α also induces cccDNA degradation by activating cytidine deaminases from the APOBEC3 family and epigenetically regulates cccDNA transcriptional activity [32,33].

Overall, HBsAg clearance and seroconversion, only occur only in 1/4 to 1/3 of patients treated with PEG-IFN-α [34,35]. This disappointing response rate may be explained by the recent findings that pharmacokinetics of PEG-IFN-α are negatively affected by the formation of PEG IFN-α-IgM complexes in the liver and their sequestration and removal by Kupffer cells [36].

PEG IFN-α treatment is also associated with frequent episodes of hepatic flares, which restricts its utility to patients with compensated liver disease [35]. It is also important to note that the considerable side effects, multiple contraindications and mode of administration (subcutaneous injections) are major issues that underpin the unpopularity of a regimen of PEG IFN-α with patients [34,35].

Given the differential mechanisms of action of NAs and PEG-IFN-α, combinatorial strategies showing some clinical benefit, are under investigation, including *de-novo* (concomitant administration of NAs and PEG-IFN-α), *add-on* (addition of PEG-IFN-α to on-going NAs) and *switch-to* (lead-in with NAs followed by PEG-IFN-α). Recent evidence suggests that sequential therapy followed by *switch-to* or *add-on* may increase combinatorial treatment efficacy in patients with suppressed viraemia and low antigenemia and those who experienced HBeAg loss on NAs [37–47]. However, more work remains to be done to determine the most beneficial combination strategy. Factors such as the scheduling of therapy, baseline HBsAg level, and suppressed viraemia may be critical in the efficacy of this type of therapy.

## Cessation of long-term NAs

Stopping NAs can be recommended in a minority (<1%) of non-cirrhotic CHB patients who have lost HBsAg. For HBeAg+ CHB patients, NAs withdrawal is recommended if there is a stable HBeAg seroconversion with undetectable viral load and at least 12 months of consolidation therapy has been achieved [5]. In contrast, the stopping rules for non-cirrhotic HBeAg- CHB patients are not well defined [48]. While some guidelines recommend indefinite NAs treatment, others recommend NA discontinuation in patients who have been treated for 2 years with an undetectable viral load documented on 3 occasions, 6 months apart. This recommendation has been tested and showed cumulative virological relapse (HBV DNA >2000 IU/mL) and clinical relapse (HBV DNA >2000 IU/mL and ALT elevation) rates of 70% and 43.6%, respectively [48]. A landmark study by Hadziyannis et al. reported sustained biochemical and virological remission in 55% HBeAg- CHB who did not require treatment due to the absence of liver disease [49]. More strikingly, 39% of these patients lost HBsAg during long-term follow-up, resulting in a functional cure. Following this report, numerous studies have assessed NAs cessation in HBeAg- CHB patients [50]. Results showed a number of patients experiencing functional cure or transitioning to the HBeAg- chronic infection stage and thus did not need further treatment. The restoration of a durable immune response has been associated with these favourable outcomes but the exact mechanisms remain to be elucidated [51–53].

There are disadvantages to this type of approach. Although generally safe, NAs discontinuation can have severe consequence such as severe ALT flares and hepatic decompensation and even death [48]. Consequently, this treatment can only be tested in a selected group of patients and demand close monitoring by an expert physician. Furthermore, due to the lack of well-defined guidelines, the benefits of this treatment need to be weighted carefully against the risks. One possible drawback is the increased risk of developing HCC due to viral rebound which occurs after NAs cessation. No increase in HCC risk has been observed so far but surveillance is recommended [54].

Identifying biomarkers that could predict successful discontinuation of NAs would be very useful and a small pilot study revealed potential immune profiles but this work requires further validation [12]. One clinical trial is exploring NAs cessation further by evaluating whether a short course of PEG IFN-α after NAs discontinuation can activate the immune response and induce the loss of HBsAg (Nucleos(t)ides withdrawal in HBeAg- hepatitis B infection to promote HBsAg clearance-NUC-B).

## Novel Direct-Acting-Antivirals (DAAs)

**Figure 1.**
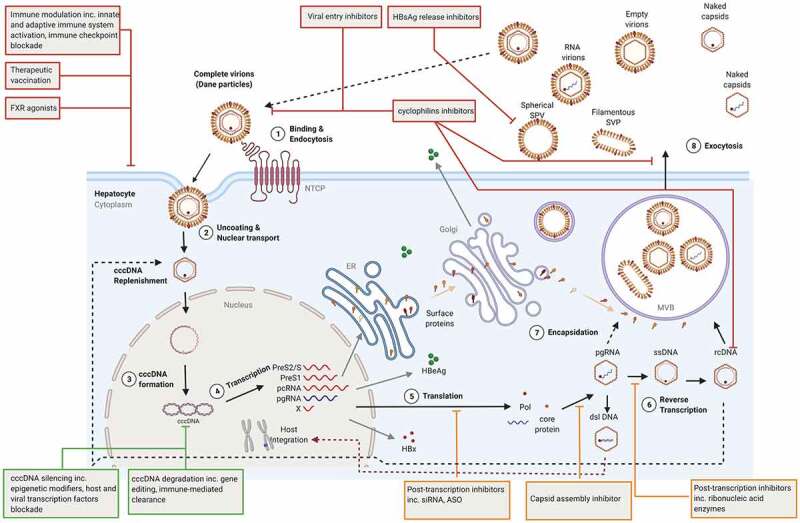
HBV life cycle and novel therapeutic targets. DAAs, HTT and cccDNA direct-acting agents are shown orange, red, and green, respectively. ASO: antisense oligonucleotides; cccDNA: covalently closed circular DNA; ER: endoplasmic reticulum; dslDna: double-stranded linear DNA; FXR: Farnesoid X receptor; NTCP: sodium-taurocholate co-transporting polypeptide; MVB: multivesicular bodies pcRNA: precore RNA; pgRNA: pregenomic RNA; Pol: polymerase; rcDNA: relaxed circular DNA, siRNA: small interfering RNA; SVP: subviral particles.

Ongoing clinical trials are evaluating new classes of DAAs to target different steps of HBV life cycle with the ultimate goal to achieve a functional cure (Figure 1). HBV core particles play a vital role in HBV life cycle, they are involved in 1) the release of the relaxed circular (RC) DNA from capsids directed to be delivered to the nucleus, 2) the packaging of the pregenomic RNA (pgRNA) for reverse-transcription into the RC DNA, 3) the composition of Dane infectious viral particles and 4) the replenishment to the cccDNA pool. As such, HBcAg constitutes an attractive antiviral target for CHB.

### Capsid assembly inhibitors

There are two main classes of core protein allosteric modulators (CpAMs):

1. Class I CpAMs known as heteroaryldihydropyrimidines (HAPS) alter the kinetic of capsid formation to produce morphologically aberrant non-capsids polymers and result in the depletion of HBV core proteins.
2. Class II CpAMs, also known as phenylpropenamides (PPA) and sulfamoylbenzamides (SBA), accelerate capsid assembly, prevent pgRNA encapsidation and lead to morphologically normal capsids devoid of nucleic acid content [55–57].

HAPS and SBA are two of the most studied CpAMs. However, novel structural classes such as sulfamoylpyrroloamides (SPAs), glyoxamoylpyrroloxamides (GLPs) and dibenzo-thiazepine-2-carboxamide derivatives (BTS) have been developed [58].

RO7049389, GLS4 and Bay41–4109 are representatives of the HAPS family. RO7049389 is a small oral molecule recently evaluated in a multi-centre, randomised placebo-controlled phase I study. Treatment with RO7049389 led to a 2.7–3.2 and 2.1–2.5 log_10_ decline in median HBV DNA and HBV RNA, respectively [59–61]. There was, however, no change in HBsAg levels and viral rebound to pre-treatment level was observed post-treatment. RO7049389 is currently under clinical evaluation with TLR7 agonist RO7020531 and NAs. GLS4 was tested in a multiple ascending dose study and, more recently, in a double-blind, randomised phase 1a study [62,63]. Given in combination with ritonavir, a metabolic enzymes inhibitor, which increased GLS4 plasma concentration, 28 days GLS4 treatment induced a less pronounced decline in HBV DNA, HBsAg and HBeAg compared to ETV alone (HBV DNA: 1.42–2.14 vs 3.5 log_10_ IU/mL; HBsAg: 0.06–0.14 vs 0.33 log_10_ IU/mL; HBeAg: 0.25–0.3 vs 0.43 log_10_ IU/mL). In contrast, the decline of pgRNA and HB core related Ag (HBcrAg), 2 biomarkers of cccDNA transcriptional activity, was greater in the GLS4+ ritonavir combination (pgRNA: 0.75–1.78 vs 0.96 log_10_ copies/mL; HBcrAg: 0.23–0.5 vs 0.44 log_10_ U/mL). However, post-treatment viral rebound was detected, and pgRNA levels returned to baseline levels. Bay41–4109 has been tested only in preclinical studies and shown to prevent the assembly and induce the destabilisation of capsids in hepatoma cell lines and humanised mice. In primary hepatocytes, Bay41–4109 reduced HBV replication, intracellular HBV RNA, antigenemia and inhibited cccDNA formation [64–67].

NVR 3–778 and JNJ6379 are two SBA molecules currently under investigation [68,69]. NVR 3–778 was among the first molecule of its class to be tested in humans. Proof of principle studies in a humanised mouse model showed that NVR 3–778 plus PEG-IFN-α could induce greater suppression in viraemia and HBV RNA [70]. The superiority and safety of this combination were later confirmed in a 28-day dose-ranging phase 1b trial with a 1.97 log10 IU/mL and 2.09 log10 copies/mL decline in HBV DNA and HBV RNA, respectively [71]. However, HBsAg, HBeAg and HBcrAg did not significantly change within the 28 days treatment across all cohorts.

In a small double-blind study that also included healthy individuals, the administration of JNJ6379 monotherapy resulted in a 2.16–2.92 log_10_ IU/mL decline in viraemia and a 1.43–2.58 log_10_ copies/mL reduction in HBV RNA [72]. Unfortunately, HBV DNA and RNA gradually returned to baseline levels after treatment cessation and no significant change in HBsAg was detected. JNJ6379 is currently evaluated in combination with NAs in a larger cohort. Despite its significant inhibitory activity against cccDNA *in-vitro*, JNJ6379 demonstrated no effect on cccDNA *in-vivo* [68].

ABI-H0731 (Verbicovir), a first-generation capsid inhibitor, has also been evaluated in combination with NAs. First results showed profound viral suppression with some patients meeting treatment stopping criteria, i.e. a decline in HBV DNA and pgRNA <20 U/mL, HBeAg seroconversion or HBeAg <5IU/mL for at least 6 months. However, in this group of patients, ABI-H0731 failed to demonstrate a sustained durable virological response off-treatment, and thus this clinical trial has been terminated [73]. ABI-H0731 is now redirected to be tested in combination therapies.

Many more CpAMs are currently in clinical development as single or in combination therapies [67,72,74,75] (see Table 4).

**Table 4.** Summary of novel antiviral therapies for CHB in clinical trial: (a) Single therapies; (b) Combination therapies. CI: Capsid inhibitor; Cyp I: cyclophilins inhibitors; HF: Host factors; Janssen Research & dvp: Janssen Research & development; Nivo: Nivolumab; NMAb: Neutralising monoclonal antibodies; NAPs: nucleic acid polymers; mAb: monoclonal antibodies; PEG: PEG-IFN-α; Pharm.: Pharmaceutical; TV: therapeutic vaccines; TLRa: TLR agonists; VIR Biotech: VIR Biotechnology. Clinical trials in the recruitment, active or not yet in the recruitment phase are highlighted in blue, green and red respectively. completed trials are excluded.

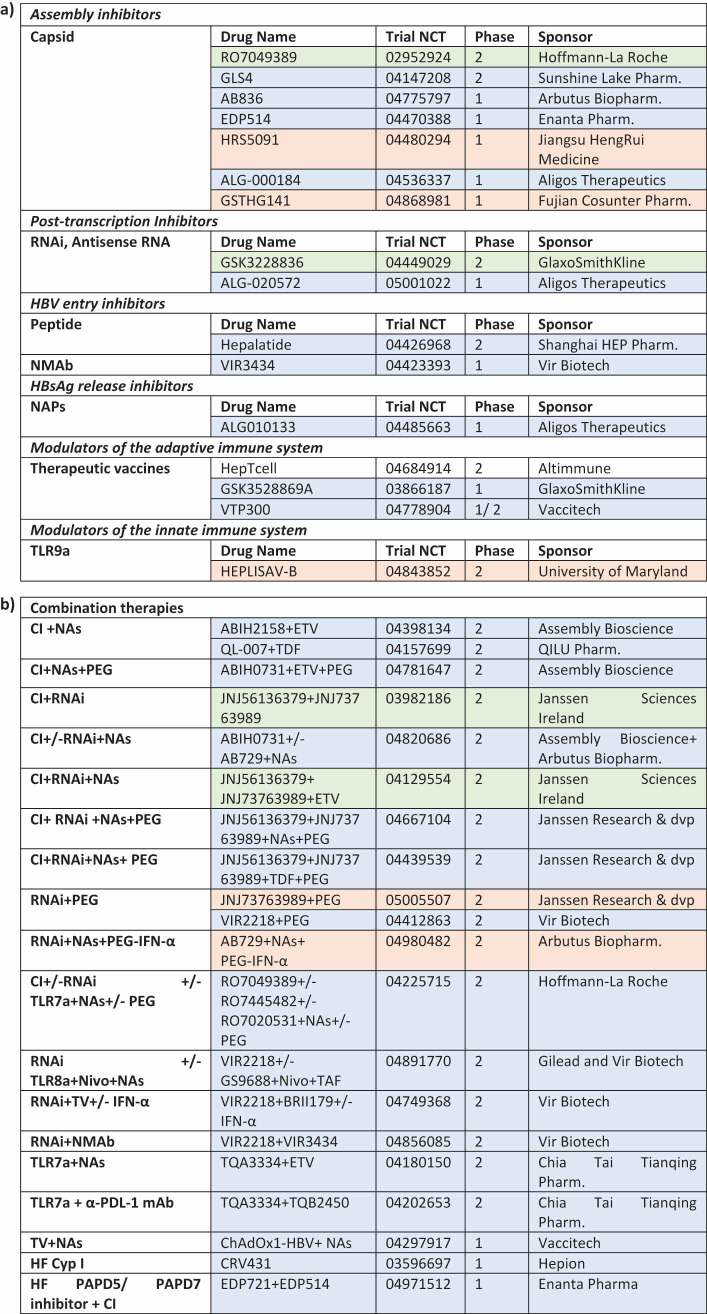

### Post-Transcriptional control

Targeting the production of HBV mRNA has emerged as a new therapeutic approach for CHB, made especially attractive by the prospect of inhibiting all five HBV mRNA transcripts with a single molecule [76]. These HBV mRNA inhibitors act by degrading mRNA or suppressing their translation to limit HBV protein production, such as HBsAg, HBeAg and HBcrAg, thereby effectively terminating active replication and the release of viral/subviral particles. Several post-transcriptional control strategies have been proposed, including RNA interference (RNAi), antisense oligonucleotides (ASO) and ribonucleic acid enzymes.

The RNAi method involves utilising 20–25 base pairs small RNAi (siRNA) molecules designed to bind to overlapping regions coding for multiple mRNA transcripts. RNAi therapeutics was initiated in a phase II clinical trial with the evaluation of ARC-520, which is composed of two cholesterol-conjugated siRNA, siHBV-74 and siHBV-77, combined with *N*-acteylgalactosamine (NAG) for targeted delivery to hepatocytes. HBsAg was strongly reduced in HBeAg+ patients treated with ARC-520, but the effect was more modest in HBeAg- or NAs experienced HBeAg+ patients [77]. This difference in response was elucidated by experiments in HBeAg- chimpanzees which established that HBsAg was predominantly produced by the integrated HBV DNA lacking ARC-520 binding sites [77]. This led to the design of next-generation siRNA such as JNJ-3989 (former ARO-HBV) which target sequences upstream of these deletions. In contrast to ARC-520, JNJ-3989 was well tolerated in a dose-ranging phase 2 study. HBsAg declined >1 Log IU/mL in 39/40 patients, and a sustained HBsAg reduction was observed in 56% of patients 9 months after the last dose [78]. JNJ-3989, coupled with TDF or ETV, induced a 0.73–3.84 Log IU/mL HBsAg reduction in 98% of patients and a sustained reduction in HBV RNA, HBeAg and HBcrAg [79].

Preliminary safety and pharmacokinetic data for repeat dosing of NAG siRNA AB-729 given to NAs suppressed CHB patients in a phase 2 study were recently presented. HBsAg declined significantly and remained <100 IU/mL in 70% of patients. Interestingly, no HBsAg rebound was detected post-treatment [80]. In a parallel study, AB-729 administered as single or repeat dosing (6/7 doses) showed total HBsAg (large, middle and small HBsAg isoforms) decline, which correlated with reduction of HBV RNA and large and middle HBs proteins irrespectively of dosing regimen [81]. Furthermore, this change in HBsAg was accompanied by an increase in HBV-specific T cell proliferative capacity and the frequency of IFN-γ-producing HBV-specific T cells, which are central to the control of HBV infection. These immunological events occurred before or during mild to moderate ALT flares [82]. AB-729 was also given as a single dose to HBeAg- participants with low viraemia and, therefore, not eligible for standard of care. It led to significant HBV DNA and HBsAg decline and undetectable HBV RNA and HBcrAg in all patients up to 36 weeks after dosing [83].

VIR-2218, an HBx targeting RNAi, was evaluated in a small ranging dose phase 2 study. The latest data showed a dose-dependent reduction in HBsAg, with levels dropping >1 Log IU/mL in 71% of HBeAg+ and HBeAg- CHB patients, but the proportion of patients reaching a sustained response was low and concerned only 4/20 patients. These results supported the development of VIR-2218 with PEG-IFN-α. Interim analysis shows that co-administration of these two drugs induced a quicker and more profound HBsAg decline than either drug alone [84].

The mode of action of antisense oligonucleotides (ASO) such as RO-293, GSK -33389404 and GSK -3228836 differ from siRNA. ASO bind to complementary sequences of HBV mRNAs and form hybrids DNA: RNA or RNA: RNA duplexes, resulting in the degradation of the RNA target via RNase-H dependent pathways. GSK -33389404, which exerted minimal efficacy, was replaced by GSK3228836, recently evaluated in a small phase 2a study. Interestingly, GSK3228836 showed at least a 3 Log_10_ IU/mL reduction in HBsAg levels in treatment naïve and experienced CHB patients and is presently moving forward in a phase 2b study.

Ribonucleic acid enzymes catalyse the cleavage of DNA: RNA complexes. HBV RNase H, for example, degrades pgRNA during the minus DNA strand synthesis within the nucleocapsids. Thus, RNase H inhibition would cause the accumulation of DNA: RNA complexes and stop the reverse transcription process, resulting in the production of defectious, non-infectious virions. However, RNase H inhibitors have yet to reach clinical evaluation because of technical difficulty associated with the production of active enzymes.

## Host-targeted therapies (HTT)

Like many other infectious agents, HBV heavily relies upon cellular host factors for almost every step of its replicative life cycle [85,86]. Furthermore, unfavourable virus-host interactions are at the heart of immune escape and the establishment of HBV persistence. Thus, targeting host factors implicated in HBV replicative cycle and activating those with anti-HBV functions are potentially valuable therapeutic approaches. Notably, as they do not add selective pressure directly to the virus but act on the host, these factors make attractive targets by offering a high barrier to resistance with a potentially pan-genotypic antiviral effect. However, they do require rigorous testing for any toxic events.

### Viral entry inhibitors

The recent identification of liver-specific bile acid transporter, the sodium-taurocholate co-transporting polypeptide (NTCP/SCLC10A1), as a host entry factor for HBV, has opened new therapeutic avenues to block viral entry and reduce viral spread [87,88]. Bulevirtide, formerly known as Myrcludex, consist of the preS1 domain of the large surface protein, which inhibits NTCP and prevents viral entry. In phase 2 dose-ranging study, Bulevirtide monotherapy was evaluated in HBeAg- CHB patients [89]. Although HBV DNA declined by > 1 Log in 32% of patients, HBsAg was not affected. As a result, most studies are focused on co-infection with hepatitis D (HDV), a satellite virus of HBV that depends on HBsAg for infection. The latest interim results showed that Bulevirtide in combination with PEG-IFN-α induced a steep decline of HDV RNA to undetectable level with some patients experiencing sustained undetectable HDV RNA 6 months after completing treatment. These results led to Bulevirtide approval for the treatment of chronic HDV by the European Medicine Agency [89–92].

HBV entry can also be interrupted by small compounds such as cyclophilins inhibitors and neutralising monoclonal antibodies (NMAb). Cyclosporine A (CsA), a well-known immunosuppressant, can prevent HBV attachment to NTCP without interfering with NTCP bile acid transport functions in experimental models [93,94]. CsA derivatives lacking immunosuppressive activities have since been developed and include SCY446, CCY450, and 27A. They can prevent HBV engagement with NTCP with great efficacy *in-vitro*, but they have yet to be pushed forward into clinical development for CHB [95,96].

VIR-3434, a pre-S1-specific NMAb, is currently evaluated in a phase 1 study [97]. Preliminary data in a small cohort of CHB showed a mean reduction of 1.3 Log IU/mL in HBsAg levels. A combination phase 2 study with RNAi VIR-2218 is in the recruitment phase.

While HBV entry inhibitors have their place in HBV therapeutic arsenal, due to their capabilities to protect naïve hepatocytes from infection, they do not directly target and nor deplete the cccDNA reservoir. Thus, the clinical value of entry inhibitors in the context of a cure for CHB may lie in combinatorial regimens aimed to prevent *de-novo* cccDNA formation and/or cccDNA degradation.

### HBsAg release inhibitors

HBsAg plays a major role in HBV life cycle. Embedded in a lipid bilayer, HBsAg forms the surface of the viral genome-containing HBV virions and allow viral entry via the binding of the preS1 region to the NTCP receptor. HBsAg also assembled around newly formed nucleocapsids for viral egress and is secreted in excess as non-infectious empty subviral particles (SVPs). As such, HBsAg is the most abundant HBV antigen accounting for 99.99% of circulating SVPs. It has long been known that this excess of HBsAg contributes to the suppressive immune environment that exists during CHB. Therefore, the appeal in blocking HBsAg release resides in the potential to prevent the release of enveloped viruses, spread of infection and restore an effective HBV-specific immune response that is able to control cccDNA and induce viral clearance. DNA based nucleic acid polymers (NAPs) (REP-2055 and REP-2031) or RNA-based NAPs (REP-2139 and REP-2165) are single-stranded nucleotides. Their antiviral functions are independent of their sequence but highly depend on their length and amphipathic nature. NAPs block the release of HBsAg, interrupting the assembly or the secretion of SVPs via its interaction with unknown host factors. Small proof-of-concept clinical trials have shown that REP-2139, REP-2165 and REP-2055 can prevent HBsAg release [98–100]. In a recent small clinical trial (REP 301/REP 301-LTF), REP-2139 monotherapy followed by a combination regimen with PEG-IFN-α followed by PEG-IFN-α alone in HBV/HDV co-infected, resulted in a rapid decline in HBsAg and an increase in anti-HBsAg Ab titres in 42% of patients which was maintained more than a year post follow up [98]. This trial also reported symptoms of heavy metal intoxication in patients, which was linked to plasma and liver accumulation of REP-2139. This has led to the design of the next generation of NAPs, REP-2165, with reduced liver accumulation properties and comparable anti-HBsAg effect to REP-2139. A phase 2 pilot study (REP 401) combining REP-2139 or REP-2165 with TDF and PEG-IFN-α was also recently investigated. At the end of treatment, 60% of HBeAg- CHB patients achieved HBsAg ≤0.05 IU/mL, all experienced HBsAg seroconversion and response towards REP-2139 and REP-2165 were similar [101]. During 48 weeks of follow-up, virological control and functional cure occurred in 32.5% and 35% of patients, respectively. Promisingly, in HBeAg- CHB patients, HBsAg produced by integrated HBV DNA is lost, suggesting that REP-2139 and REP-2165 can reduce integrated DNA, but the mechanism has not been investigated. Additionally, more than 90% of patients experienced ALT flares which were more pronounced in those who lost HBsAg suggesting host-induced flares representing immune control of infection were at play. Subsequent analysis of virological markers in REP 301, REP 301-LFT and REP 401 trials, presented recently, showed a functional cure could be reached in most patients and was characterised by undetectable HBV DNA and HBV RNA, HBcrAg below the lower limit of quantification, normalised ALT levels, HBsAg decline below 0.005 IU/mL, and the detection of HBsAg Ab [102]. These impressive results need, however, to be validated in larger studies.

*In-vitro* studies which confirmed HBsAg release blockade by NAPs also revealed a mild entry inhibitory activity which is thought to be mediated by the interference of HBV binding to heparan sulphate proteoglycans [103,104]. However, this latter function does not concern the latest compounds REP-2139 or REP-2165, which primarily affect the formation and secretion of SVPs. It is also thought that NAPs can prevent virus egress and also promote anti-HBV immunity, although these mechanisms need to be confirmed better [105]. Indeed, induction of cytokines production from healthy individuals’ peripheral blood mononuclear cells treated with a high dose of NAPs was observed. In contrast, primary hepatocytes and liver sinusoidal endothelial cells were resistant to NAPs immunomodulatory effects, suggesting that NAPs antiviral activity in patients could not be attributed to the induction of innate antiviral responses. Altogether, NAPs molecular modes of action remain unknown.

### Farnesoid X receptor (FXR) agonists

HBV cccDNA regulation is modulated by multiple transcription factors. Among these factors, the bile acid nuclear receptor FXR binds to two response elements on cccDNA and induces HBV transcription. FXR is also involved in the synthesis and maintenance of cccDNA. The proviral activities of FXR can be interrupted by its engagement with agonists, which reportedly inhibits cccDNA transcription [106,107]. The mechanisms have yet to be fully elucidated, but it is thought that FXR agonists may disrupt the active form of FXR, alter FXR function and/or may destabilise the interaction between FXR and other cellular factors involved in pgRNA transcription [106]. Interestingly, several studies have provided evidence that FXR can also promote cccDNA transcription. A phase 2 study is evaluating FXR agonist EYP001 (Vonafexor) with PEG-IFN-α and ETV in treatment naïve HBeAg+ and HBeAg- CHB [108]. The interim results showed a profound HBV DNA and HBsAg decline in the HBeAg+ group but less of a response in the HBeAg- (−3.7 vs −0.4 Log IU/mL; −0.9 Log vs −0.0 IU/mL). HBsAg declined more steeply in the EYP001 plus PEG-IFN-α group over the EYP001 with PEG-IFN-α and ETV. ALT/AST flares were experienced but resolved upon treatment interruption.

### Cyclophilins inhibitors

Cyclophilins are host proteins that belong to the large family of immunophilins and exert peptidyl-prolyl isomerase (PPIase) enzymatic activities. They catalyse the conversion of X-Prolyl bonds (× represents any amino acid) from cis to trans conformation, participating in protein folding [109]. Cyclophilins are also implicated in proteins trafficking, cellular signalling and immune modulation. The human genome encodes for 16 cyclophilins isoforms which are located in various cellular compartments, of which eight can be secreted [110]. Several cyclophilins, including cyclophilin A, B and 40, are upregulated and implicated in multiple pathologies, including cancer. A large body of evidence has confirmed that cyclophilins participate in human immunodeficiency virus (HIV) infection and replication [111–120]. PPIase activity was also found involved in HCV replication cycle and led to the development of a series of cyclophilin inhibitors such as NIM811, DEBIO-025 (Alisporivir) and SCY-635, which have demonstrated anti-HCV activity in patients in the absence of toxicity, identifying cyclophilins as therapeutic target for chronic hepatitis C [121–126]. Unfortunately, cases of severe pancreatis were reported with one death which led to the compound clinical testing to be discontinued. All cases of pancreatis occurred in the PEG-IFN-α/HCV antiviral ribavirin triple combination but not with DEBIO-025 alone. Latter findings demonstrated that these serious adverse events were not related to DEBIO-025.

Cyclophilins are also implicated in HBV life cycle. *In-vitro*, DEBIO-025 impedes viral replication rates and slow HBsAg production [127]. Knockdown experiments revealed that cyclophilin A and, to a lesser extent, cyclophilin C and D are actively involved in the HBV life cycle and that DEBIO-025 had pan-cyclophilin blocking properties [127]. The antiviral effect of cyclophilin blockade was also confirmed *in-vitro* and in mice with NVP018, a Sangamide cyclophilin inhibitor [128]. To date, a second generation of cyclophilin inhibitor CRV431 showed great efficacy in mouse models of HBV infection without cytotoxic effects and is currently under investigation in healthy subjects [129]. CRV431 is also currently under evaluation for non-alcoholic steatohepatitis (NASH) and HCC [130,131]. Results from preclinical studies in animal models of NASH, in human cells cultures and tissue explants revealed CRV431 antifibrotic efficacy and supported CRV431 evaluation in a phase 2a clinical trial.

### Directly targeting cccDNA

HBV cccDNA is the barrier to HBV cure [132]. This highly stable mini-chromosome is protected within the nucleus of hepatocytes and drives the continuous transcription of viral proteins. Therefore, drugs targeting cccDNA may achieve a functional and complete cure. As a result, treatments designed to silence or destroy cccDNA have gained much traction in recent years. However, targeting cccDNA remain a major challenge, and consequently, there are no therapeutic targets in clinical development.

The transcription of HBV cccDNA is regulated by numerous host factors and by viral proteins such as HBx and HBcAg. Several reports have described the involvement of HBx protein in regulating cellular pathways, transcription factors and promotion of liver tumorigenesis [133]. A recent study reported that HBx binding to the damaged-specific DNA binding protein 1 (DDB1) degrades the ‘structural maintenance of chromosomes (Smc) complex SMC5/6, a restriction factor capable of blocking HBV RNAs transcription [134]. Interestingly, Nitazoxanide, an anti-parasitic drug, was shown to stop the degradation of this SMC5/6 complex and consequently inhibit HBV replicative intermediates, including cccDNA *in-vitro* [135]. In a pilot study, Nitazoxanide was administrated for up to 48 weeks to 9 treatment naïve CHB participants [136]. The drug was overall well tolerated, with side effects ranging from mild to moderate. Interestingly, HBV DNA became undetectable, and HBsAg was lost in 89% and 33% of participants, respectively. In addition, HBeAg seroconversion occurred in the 2 HBeAg+ subjects. At the end of the study, all patients were given NAs as per the study protocol and this did not allow a follow up of Nitazoxanide antiviral effect post-treatment. To date, there has been no further clinical investigation of Nitazoxanide as a therapy for CHB.

Given its chromosomal organisation, cccDNA associates with histones and non-histone proteins, including proteins of viral and host origins. Many of these proteins are recruited to regulate the HBV mini-chromosome at an epigenetic level [137,138]. For example, following its recruitment onto cccDNA, HBx increases the expression of DNA methyltransferase (DNMTs) while HBcAg interacts with histone acetyltransferases (HATs) to promote cccDNA transcription [138,139]. Therefore, targeting pro-HBV epigenetic changes with the help of epigenetic modifiers could promote cccDNA silencing. Several epigenetic drugs targeting DNA methylation or histone acetylation are in development for treating cancers, including HCC and could be repurposed for CHB [140,141]. For example, AGK2, a histone deacetylase Sirtuin 2 inhibitor, was shown to repress HBV replication in cell lines and transgenic mice [142,143]. GS-5801, a lysine demethylase inhibitor, showed great efficacy against HBV in primary hepatocytes but no effect in a phase 1 study and was discontinued [144]. However, despite its appeal and strong potential, epigenetic therapy can induce unwanted side effects due to the involvement of epigenetic enzymes in cellular processes. Thus, targeting the mechanisms of HBx and HBcAg-mediated cccDNA regulation may be a safer alternative approach. However, in order to do so, the elucidation of the molecular mechanisms regulating cccDNA biosynthesis, transcription and turnover is paramount.

APOBEC3 (A3) enzymes can directly target cccDNA. Their function is to deaminate cytidines into uridines on cDNA during reverse transcription, leading to G-to-A hypermutation upon second DNA strand synthesis. The A3 family is composed of 7 deaminases (A3A, A3B, A3C, A3D, A3F, A3 G and A3 H) and plays an important role in the innate immune defence mechanisms. For example, A3 G restricts HIV-1 infectivity by inducing the degradation of the hypermutated viral genome [145–147]. Recent studies have shown that IFN-α, IFN-β, and IFN-lambda (IFN-λ) can upregulate A3A and A3 G and trigger the degradation of cccDNA [148]. Similarly, IFN-γ and TNF-α produced by HBV-specific T cells led to cccDNA degradation via A3-induced deamination [149]. However, only partial cccDNA clearance was achieved in these studies.

Genome-editing technologies such as Zinc finger nuclease (ZFN), transcription activator-like effector nucleases (TALENs) and more recently, clustered regularly interspaced short palindromic repeats (CRISPR)/CRISPR-associated 9 (Cas9) have the potential to stop the formation of cccDNA and limit its accumulation in the nucleus. These editing systems create DNA double-strand brakes (DBS), which activate the erroneous repair of cccDNA by nonhomologous end-joining pathways, induce the formation of insertions and deletions, leading to the disruption of the open reading frames of genes. Interestingly, gene editing can also cause the degradation of cccDNA [150,151]. Numerous preclinical studies have shown that CRISPR/Cas9, the most efficient of these platforms, can successfully cleave and inactivate cccDNA in hepatoma cell lines and hydrodynamically injected mice [152–158]. However, concerns over genomic instability due to host DNA cleavage and off-target effects and challenges such as high HBV genomes heterogeneity, lack of delivery specificity, and pre-existing immunity to Cas9 have prevented gene editing from reaching clinical development [159–161]. More work is currently underway to overcome these limitations [156,162,163]. Interestingly, a novel CRISPR-derived’ base editors’ technology is exploring the introduction of point mutations in cccDNA and integrated HBV DNA fragments without inducing DBS [164].

More research needs to be done to increase our understanding of cccDNA biology and advance therapeutic strategies with curative potential.

### Immune-Based therapies

**Figure 2.**
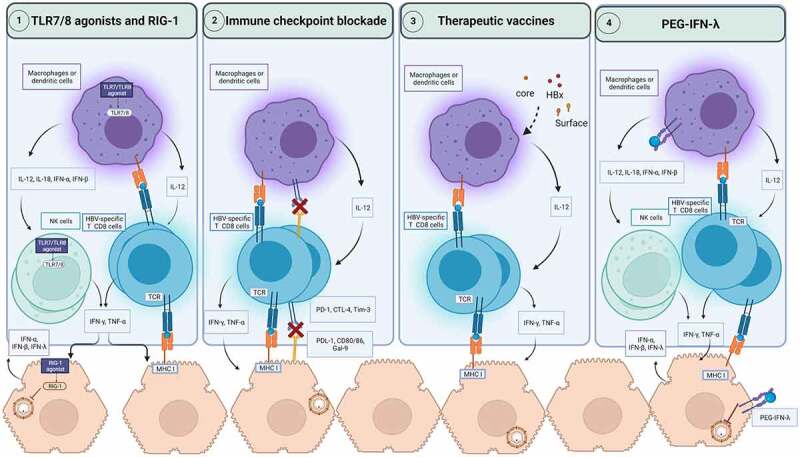
Immune based therapeutic strategies for CHB. The activation of pathogens recognition receptors such as TLR7, TLR8 and RIG-1 by agonists can induce the secretion of antiviral cytokines by hepatocytes and innate immune cells such as macrophages, dendritic cells, and NK cells. This activation of innate immunity can restore HBV-specific T cells to produce antiviral cytokines such as IFN-γ and TNF-α. RIG-1 can also exerts direct antiviral activities against HBV. Immune checkpoint blockade prevents the engagement of inhibitory checkpoints express on HBV-specific T cells with their ligands on macrophages, dendritic cells, and hepatocytes. As a result, HBV-specific T cells functionality can be restored to produce antiviral cytokines and potentially cytotoxic response to clear infected hepatocytes. Therapeutic vaccines encoding for viral antigens, can enhance cross-presentation of these antigens and boost HBV-specific T cell response. PEG-IFN-λ therapy can modulate dendritic cells, and macrophages and mediate the cross talk with NK and HBV-specific T cells. In hepatocytes, PEG-IFN-λ induces the engagement of signaling pathways which suppress HBV replication. CTLA-4: Cytotoxic *T*-lymphocyte associated protein 4; IFN: Interferon; IL: Interleukin; MHC: Major histocompatibility complex; NK cells: natural killer cells; PEG-IFN-λ: Pegylated interferon-lambda; PD-1: Programmed death pathway-1; RIG-1: Retinoic acid-inducible gene; Tim-3: T cell immunoglobulin and mucin-domain containing-3; TLR: Toll-like receptor; TNF-α: Tumor necrosis factor-alpha.

Lessons from natural, spontaneous resolution of HBV infection have revealed that the orchestration of a strong and coordinated response from the innate and adaptive arms of the immune system is vital for long-term durable control of HBV infection [10,11,165]. In contrast, the progression to CHB stems from a dysfunctional and exhausted antiviral immune response [165–168]. Therefore, harnessing the immune system to restore the impaired HBV-specific immunity provides the basis for developing immunomodulatory therapeutic strategies (see Figure 2) [169].

#### Activation of the innate immune system

HBV can be recognised by pathogens recognition receptors (PRRs), including Toll-Like Receptors (TLRs) and Retinoic acid-inducible gene 1 (RIG-1) [170–172]. Each PRRs engagement leads to the activation of specific downstream signalling events resulting in local IFN type I and type III responses and other pro-inflammatory cytokines and chemokines, which may trigger the activation of NK cells and promote the restoration of the HBV-specific adaptive immune response [170–172]. The virus, however, has devised multiple ways to inhibit PRRs signalling pathways to escape immune surveillance [173]. Thus, pharmacological stimulation of PRRs is thought of as a strategy to reactivate both the innate and adaptive arms of the immune response for viral clearance [174].

##### Tlrs

TLR7 and TLR8 are endosomal sensors of single-stranded RNA molecules expressed by haematopoietic cells, such as B cells, monocytes/macrophages and dendritic cells, NK cells, and cytotoxic T cells [175–179]. Several TLR7 (RO7020531, JNJ-4964, GS-9620) and TLR8 (GS-9688) agonists are currently under evaluation. RO7020531 was well tolerated with a good safety profile in healthy participants and has now moved onto a combination regiment with capsid inhibitors RO7049389 in CHB patients [180]. GS-0620 (Vesatolimod) induced a strong antiviral effect in woodchuck and chimpanzees [181,182]. However, despite the strong preclinical debut of GS-0620 and a noticeable increase in NK cells and HBV-specific T cells, this was not accompanied by a significant decline in HBsAg [183,184]. One possible explanation for this loss in the efficacy of GS-0620 is the lower dosage given in patients compared to chimpanzees (4 mg per patient versus 1 mg/kg in chimpanzees) to minimise possible toxic events. TLR7 agonist JNJ-4964 was tested in AAV-HBV mice for 12 weeks and induced a strong suppression of HBV DNA and HBsAg levels and a sustained production of HBsAg [185]. JNJ-4964 was also tested in healthy individuals and was well-tolerated as a single dose and induced transient production of IFN-α, IP-10, MCP-1, IL-1RA and ISGs [186]. TLR8 agonist GS-9688 (Selgantolimob) was given at 1.5 mg or 3 mg to virally- suppress HBeAg+ CHB [187,188]. The latest data showed that no patients achieved ≥1 Log IU/mL decline in HBsAg and only 6% achieved ≥0.5 Log IU/mL at the end of treatment. Furthermore, HBeAg seroconversion occurred only in 16% of HBeAg+ patients. Immunological analysis showed a transient, dose-dependent induction in circulatory IFN-γ in patients treated with GS-9688 while the frequency of circulatory CD3+ T cells was reduced, probably due to T cell relocation in the liver. GS-9688 was also tested in combination with TAF and showed a reduction in HBsAg >0.3 Log IU/mL. *In-vitro* experiments showed that stimulating healthy and CHB PBMCs with GS-9688 activated NK, HBV-specific T cells, and mucosal-associated invariant T cells (MAIT) but reduced T regulatory cells, and monocytic monocytes derived suppressor cells (MDSCs), two cell subtypes known for their immunosuppressive properties. Conversely, GS-9688 increased the immunosuppressive functions of MDSCs, which may explain its limited efficacy in some patients [189].

##### Retinoic acid inducible gene 1 (RIG-1)

RIG-1, an intracytoplasmic double-stranded RNA sensor, is also a target for pharmacological activation of the immune response to CHB [172]. Like TLRs, once activated, RIG-1 leads to signal transduction through intracellular signalling pathways, which induce the production of IFN and other cytokines. Additionally, a recent study reported that the epsilon encapsidation signal found in pgRNA could be recognised by RIG-1, promoting the production of type III IFN rather than type I [190]. This induction of type III IFN also known as IFN-λ, suggests potent antiviral activity as this cytokine is known for directly inhibiting HBV replication and activating innate and adaptive immunity in CHB patients [191–193]. Additionally, RIG-1 can interfere with the epsilon-HBV polymerase interaction, directly suppressing HBV replication. Therefore, RIG-1 seems to exert a dual role as a modulator of an innate immune response and a direct antiviral effector against HBV. RIG-1 agonist SB-9200 (Inarigrivir) was given as a monotherapy followed by a switch to TDF in HBeAg+ and HBeAg – CHB patients. HBV DNA and RNA were reduced in both groups in a dose-dependent manner, while HBsAg was reduced by >0.5 Log in 22% of patients. In comparison, a dose-ranging study with TAF showed no dose-dependent changes in viral load and HBsAg [194]. However, all trials of SB-9200 were terminated due to unexpected serious adverse events, including hepatocellular dysfunction and elevation in ALT in 3 patients and 1 death in the phase 2 CATALYST trial. The mechanisms underlying these severe adverse events are currently being investigated. This once more highlights the importance of safety in the development of new therapeutic drugs for CHB.

Of note, one immune evasion mechanism employed by HBV is the suppression of PRRs expression on hepatocytes, Kupffer cells and haematopoietic cells, which may explain the reduced efficacy of PRR agonists in CHB [195,196]. Encouragingly, recent studies have reported that antivirals, more specifically PEG-IFN-α, can restore TLRs expression, and this may support the idea of combination strategies with PEG-IFN-α [197].

##### Immune checkpoints blockade

Immune checkpoints receptors (ICRs) are master regulators of the immune system [15,198]. Inhibitory and stimulatory ICRs are needed to maintain self-tolerance, prevent autoimmunity and orchestrate an adequate immune response to infections but also malignancies. The manipulation of ICRs, more specifically blockade of inhibitory checkpoints (ICs), has revolutionised cancer therapy in the past few years. In the context of CHB, which is characterised by high levels of antigens and pro-inflammatory cytokines, programmed-death-1 (PD1), cytotoxic-associated antigen 4 (CTL4), and T cell immunoglobulin and mucin containing-3 (Tim3), to name a few, chronically suppressed immune functions and promote persistence of HBV infection. The overexpression of these ICs favours a paradigm whereby the active immune system induces pathogenesis and preserve the functional and structural integrity of organs but fails at mounting an effective HBV-specific response. A study by Evans et al. was amongst the first to show that in CHB, PD1 upregulation was associated with HBV-specific T cell dysfunction [15]. Furthermore, PD1 correlated with viraemia and HBeAg and decreased progressively with NAs treatment and even more so during HBeAg seroconversion. Thus, ICs blockade may reverse HBV-specific immune dysfunction and restore an immune response capable of clearing CHB.

So far, studies evaluating anti-PD1 blockade therapy have shown disappointing results. In a small study assessing low-dose of Nivolumab in virally suppressed HBeAg- CHB, a functional cure was achieved in only 1 out 14 patients, and only a minimal decline in HBsAg was observed [199]. The treatment, however, was well tolerated. Immunological assessment was pursued and showed no changes in the T cell response over time. Considering the widespread IC dysregulation on immune cells and the wide variety of these molecules, it should not be surprising that blockade of single ICs resulted in limited efficacy. Higher dosage, simultaneous blockade of other ICs and combination therapy with novel compounds could be strategies worth considering to promote T cell restoration. However, one must be mindful of unleashing a strong immune response and inducing hepatotoxic events which have been widely reported as severe adverse events associated with ICs blockade during anticancer immunotherapy [200,201].

#### Direct activation of the adaptive immune system

##### The interferon system

As discussed earlier, PEG-IFN-α has been relegated as a second-line treatment due to its association with severe side effects and limited efficacy in patients. However, despite these shortcomings, as the only approved drug with a finite duration, PEG-IFN-α is now coupled with antiviral agents in clinical development.

IFN-λ, the latest addition of the IFN family, has been considered a better alternative for CHB [202]. IFN-α and IFN-λ share many properties. They can be induced in response to viral infections and activate an antiviral immune response via the engagement of signalling pathways such as JAK-STAT and the induction of IFN-stimulated genes. The main distinction between the two cytokines lies in their receptors’ distribution, which governs the tropism of these cytokine-induced immune responses. While IFN-α receptor is ubiquitously expressed, IFN-λ‘s is more restricted to epithelial cells and immune cells and thereby is associated with lesser side-effects compared to IFN-α.

PEG-IFN-λ was evaluated in two studies. In a head-to-head comparison with PEG-IFN-α (LIRA-B 2a) in HBeAg + CHB patients, PEG-IFN-λ1a showed deeper declines of viral load and HBsAg on-treatment and HBeAg seroconversion was equal in both arms of the study [192]. However, PEG-IFN-λ was not superior to PEG-IFN-α 24 weeks post-treatment but demonstrated a superior safety profile due to its receptor restricted distribution. The immunological and molecular mechanisms underlying this differential effect is unknown and merit further investigation.

A second trial with a lead-in ETV followed by ETV and PEG-IFN-λ combination was conducted in parallel (LIRA-B2b). Longitudinal immunological surveillance was performed and showed PEG-IFN-λ was able to promote robust NK and HBV-specific T cell functions in patients who experienced a greater decline in viraemia and antigenemia [191]. This was in contrast with PEG-IFN-α, which has a deleterious effect on T cell functions. Unfortunately, this study was prematurely discontinued due to PEG-IFN-λ failing to meet the non-inferiority criteria in the LIRA-B 2a study. PEG-IFN-λ has now been repurposed for HDV as a monotherapy or combined with a farnesyltransferase inhibitor or an antiretroviral and shows significant HDV RNA reduction in HBV/HDV co-infected patients and superior tolerability [203,204].

From these studies, we can conclude that PEG-IFN-λ can exert antiviral effects against HBV very much distinct from that of PEG-IFN-α. Understanding the mechanisms underlying these effects may help develop new ways to harness IFN-λ and overcome the limitations of this treatment.

##### Therapeutic vaccination

Therapeutic vaccines must differ from their prophylactic counterparts and restore the impaired T cell response while priming a new immune response, including the humoral arm. Strong preclinical data in animal models supported the use of therapeutic vaccines for CHB [205–207]. Unfortunately, this was not confirmed in CHB patients. For example, the administration of heat-inactivated yeast-based vaccine encoding for HBsAg, HBcAg, and HBx, GS-4774 did not result in additional HBsAg decline compared to patients who only received NAs [208–211]. Despite these disappointing results, GS-4774 increased the production of IFN-γ, TNF-α and IL-2 by HBV-specific CD8+ T cells but to level no way near the response seen in acute limited infections [210]. This limited efficacy could be explained by the high levels of HBsAg circulating in CHB patients.

ABX-203 (HeberNasvac), an intranasal vaccine containing both HBsAg and HBcAg, was tested in a phase 3 study and compared to PEG-IFN-α alone. At the end of the treatment, HBV DNA was equally suppressed in both groups, while in the follow-up phase, 57.7% of the vaccinated patients had a sustained viral load below 250 copies/mL against 35% in the PEG-IFN-α group [212]. Additionally, HBeAg seroconversion was more frequent in the vaccinated group. Unfortunately, HBsAg was not quantified, but qualitative assessment shows no loss of HBsAg in either group.

INO-1800 is a DNA-based vaccine encoding for HBsAg tested in a dose-escalating phase 1 study in mono- or combination therapy with INO-9112, which encode for IL-12 in NAs virally suppressed CHB patients. A good safety profile and improvement in the IFN-γ HBV-specific T cell response was recently announced.

The therapeutic vaccine, BRII-179, a virus-like particle encoding for all three HBsAg proteins, was recently evaluated in a phase1/2b, and multiple doses were administered with or without IFN-α in NAs suppressed CHB patients [213]. BRII-179 elicited a humoral response in all vaccinated patients, but the strongest was detected against S protein in 30% of patients who received single therapy. A more moderate response was detected against PreS1 and PreS2 but only in the IFN-α combination arm. An immunological sub-analysis was conducted in a smaller cohort. The frequency of IFN-γ-producing T cells significantly increased in 3 out of 8 patients from the IFN combination arm, but no significant changes were observed in the other study arms. Interestingly, patients who received the highest vaccination dose exhibited a more robust immune response. Despite BRII-179 activation of cellular and humoral response, there was no significant change in circulating HBsAg, HBV RNA and HBcrAg, suggesting that the induced immunity was not sufficiently potent to have an effect.

Non-replicative adenovirus, TG-1050 encodes for a fusion protein consisting of HBV polymerase, HBcAg and HBsAg. Virally suppressed CHB received either a single dose or three doses of the vaccine. TG-1050 was well tolerated, but HBsAg declines were minimal. From an immunological point of view, TG-1050 induced IFN-γ HBV-specific T cell response against all three HBV antigens.

One major disadvantage of therapeutic vaccines is the induction of an immune response against the vector backbone, which can attenuate HBV-specific responses and preclude multiple dosages. A heterologous prime-boost vaccine approach could be the answer to this problem.

## Combination therapies

Given the complexity of HBV replicative life cycle and the deep immune paralysis that characterises CHB, combinatorial therapies with agents with distinct mode of actions seem a promising approach for a cure. Synergistic effects with the standard of care, NAs and PEG-IFN-α are the most tested, but combinatorial therapies of novel drugs in clinical development are also under evaluation, with pharmaceutical companies joining forces and working together to accelerate HBV cure research (see Table 4). For example, studies testing the safety and efficacy of capsid inhibitor ABI-H0731 combined with RNAi AB-729 and TLR-8 agonist with RNAi VIR-2218 are under clinical evaluation.

A phase 2 triple combination therapy with capsid inhibitor JNJ6379, siRNA JNJ-3989, and NAs has reported a 1.01–2.26 Log IU/mL HBsAg decline in all patients accompanied by a significant reduction in HBV DNA but showed little effect on HBeAg and HBcrAg [214]. A phase I pilot study has evaluated anti-PD1 Nivolumab with therapeutic vaccine GS-4774 in HBeAg- CHB and showed a mean HBsAg decline of 0.3 Log IU/mL with a sustained loss in 1 patient [199]. Many more therapies include triple combination therapy of capsid assembly inhibitor RO7049389 with TLR7 agonist RO7020531 and NAs; HBx RNAi VIR-2218 combined with NMAb VIR-3434 or TLR7 agonist RO7020531 with RNAi RO7445482 (see Table 4).

## Conclusion and future perspectives

The development of curative treatments for CHC is the culmination of a long-term and concerted research effort. However, the challenges of curative interventions for CHB are different due to the presence of cccDNA and HBV DNA integration and will require global collaboration and creative solutions. The last few years have seen a dramatic surge in novel drug development for CHB, with multiple targets showing profound antiviral properties and taking the HBV field a step closer to achieving a functional cure. However, larger, and longer well-designed clinical trials are needed to determine the success rate of these drugs and their efficacy in inducing a long-lasting functional cure.

Given the complexity of CHB, it is likely that the combination of new agents with distinct modalities is the future of HBV therapeutics, particularly if the elimination of cccDNA is the ultimate goal. Strategies with excellent safety profiles that combine targeting the virus with DAAs and/or virus-directed HTT together with an immune-restorative approach are likely to be most successful in achieving a curative state akin to that of natural resolution. Hence, more studies are warranted to identify druggable immune mechanisms and the basic components required for HBV life cycle, more specifically involved in cccDNA biosynthesis and biology to advance new concepts into the clinical stage. It is clear that significant challenges remain, but for the first time in 20 years, there is renewed hope that a cure for CHB is insight.

## Data Availability

Data sharing is not applicable to this article as no new data were created or analysed in this study.
